# Do increasing participation rates in early childhood education narrow the reading achievement gap between high- and low-SES primary school students?

**DOI:** 10.1186/s40536-025-00274-7

**Published:** 2026-01-02

**Authors:** Isa Steinmann, Márton Medgyesi, Maria Symeonaki

**Affiliations:** 1 Oslo Metropolitan University, Oslo, Norway; 2 TÁRKI Social Research Institute, Budapest, Hungary; 3 Child Opportunities Research Group, ELTE Centre for Social Sciences, Budapest, Hungary; 4 Panteion University of Social and Political Sciences, Athens, Greece; 5 Corvinus Institute for Advanced Studies, Corvinus University, Budapest, Hungary

**Keywords:** Early childhood education, Socioeconomic inequality, Country-level trends, PIRLS, Reading achievement

## Abstract

**Background:**

Many countries around the world are expanding the enrollment of children in early childhood education (ECE) outside the home to improve school readiness and, ultimately, academic achievement. While socioeconomically disadvantaged children are typically underrepresented in ECE, they are often thought to benefit the most from participation. This study empirically tests whether increasing ECE participation rates are associated with reductions in socioeconomic inequalities in ECE participation and, consequently, with decreases in socioeconomic inequalities in reading achievement at the end of primary school.

**Methods:**

The study draws on data from 64 education systems participating in PIRLS cycles between 2001 and 2021, comprising 225 country-by-year observations. Using country-fixed effects, we estimate a mediation model to test whether socioeconomic inequalities in ECE participation mediate the relationship between overall ECE participation rates and socioeconomic inequalities in reading achievement. This fixed effects approach controls for time-invariant differences between education systems. We include a set of control variables to account for economic, demographic, and policy-related changes that might confound within-country trends and discuss untestable assumptions that have to be met to interpret the mediation relationship causally. Additionally, we examine international trends in all three variables of interest.

**Results:**

As hypothesized, we find that countries with increasing ECE participation rates and decreasing socioeconomic inequalities in ECE participation also show declining socioeconomic inequalities in reading achievement. Internationally, ECE participation rates have risen over time, while both socioeconomic inequalities in participation and socioeconomic inequalities in reading achievement did not show clear trends over time.

**Conclusions:**

According to our interpretation, increasing ECE participation rates seem to help reduce socioeconomic inequalities in reading achievement—provided that the increases also reduce disparities in access to ECE. This finding, together with previous research, offers encouraging evidence for countries investing in or planning to expand ECE. Moreover, this study highlights the importance of ensuring that ECE expansion policies specifically address socioeconomic inequalities in participation to mitigate later educational inequalities.

## Introduction

Internationally, many countries aim to expand universal early childhood education and care both in terms of overall participation rates and the number of years children attend (e.g., OECD, 2025). According to the International Standard Classification of Education (ISCED), “early childhood education” (ECE) forms ISCED Level 0—formalized educational programs outside the family prior to primary school (UNESCO, 2012). Within this level, programs for children under the age of three are classified as “early childhood educational development,” while programs for children between the age of three and the start of primary school are categorized as “pre-primary education” (UNESCO, 2012). Also, terms such as “preschool” or “pre-primary” refer to ISCED Level 0 programs (UNESCO, 2012). The term “early childhood education and care” (ECEC) is typically used more broadly to include informal and unregulated childcare settings (OECD, 2025).

The United Nations’ Sustainable Development Goal 4.2 calls for ensuring that, by 2030, “all girls and boys have access to quality early childhood development, care and pre-primary education” (United Nations, 2015, p. 21). Similarly, the European Union has set a target that by 2030, at least 96% of children aged three and older, and at least 45% of children under the age of three, should attend ECEC across member states. To achieve these goals, some EU countries have implemented policies such as making ECEC services free of charge (e.g., Portugal, Luxembourg), granting children legal entitlement to these services (e.g., Germany, Estonia), or making participation compulsory—either from age three (e.g., France, Hungary) or during the year before primary school (e.g., Finland, Greece) (European Commission: Directorate-General for Education, Youth, Sport and Culture, 2024). Nonetheless, ECEC provision policies vary widely across countries (Bendini & Devercelli, 2022; OECD, 2025).

International reports show that while participation rates tend to increase over time, there are pronounced cross-country differences in both the levels attained and, to a certain extent, the trends observed (European Commission: Directorate-General for Education, Youth, Sport and Culture, 2024; Eurostat, 2024; OECD, 2025; OECD Family Database, 2024). Across OECD countries, for example, the ECEC enrollment rates of children aged three to five range from 100% in France and the United Kingdom to rates below 30% in Turkey and South Africa (OECD Family Database, 2024). Among children under three, enrollment rates exceed 60% in the Netherlands and South Korea but remain below 10% in countries such as Czechia, Costa Rica, Indonesia, Argentina, Mexico, the Slovak Republic, and Turkey. Similarly, the average weekly hours children spend in ECEC services vary substantially across countries (ibid.).

ECEC expansion policies generally pursue two main objectives (OECD, 2025):

1. to improve the compatibility of work and family life and to keep parents, especially mothers, of young children in the workforce (e.g., Morrissey, 2017), and
2. to improve children’s school readiness and thus future academic achievement (e.g., language development, foundational mathematical skills, socio-emotional competencies) (e.g., Blewitt et al., 2018; Ulferts et al., 2019; Von Suchodoletz et al., 2023).

Regarding the educational objective of ECEC to prepare children for school, it is widely assumed that ECEC offers particular benefits for socioeconomically disadvantaged children and those who speak another language at home (e.g., Leseman & Slot, 2014; OECD, 2025). The premise is that while socioeconomically privileged children would also receive stimulating learning environments outside of ECEC services, socioeconomically disadvantaged children would receive more stimulating learning environments if they attended ECEC. For instance, two meta-analyses confirm that the effects of universal ECEC on child development are generally stronger for socioeconomically disadvantaged children (Schmutz, 2024; Van Huizen & Plantenga, 2018).^1^ A vast amount of research shows that socioeconomic disadvantages are associated with disadvantages in educational outcomes such as achievement (e.g., Korous et al., 2022; Liu et al., 2022). Discussed reasons for these disparities in education are manifold, including that disadvantaged families struggle to support children’s learning due to lower financial, social, and educational resources (e.g., Broer et al., 2019; Volante et al., 2019). Countries usually strive to minimize socioeconomic inequalities in educational outcomes, for example by expanding ECEC programs (e.g., Broer et al., 2019; Strietholt et al., 2019).

Even though countries aim to reduce socioeconomic disparities and disadvantaged children seem to profit more from ECEC participation, it is a well-established fact that socioeconomically disadvantaged children participate *less often* in universal ECEC than more privileged children in most countries (e.g., Hogrebe & Strietholt, 2016; Kulic et al., 2019; OECD, 2025; Van Lancker, 2013; Waldfogel et al., 2023). Several explanations have been proposed for this disparity, including the high costs of participation, differing parental values (e.g., mothers with higher education levels may place more importance on both ECE and their careers), and structural limitations in service availability (e.g., Pavolini & Van Lancker, 2018; Van Lancker & Ghysels, 2016; Yang et al., 2024).

In light of these findings, we assume that expanding ECEC participation has the potential to reduce later educational inequalities between socioeconomic groups—especially if the expansion also leads to a narrowing of participation gaps (cf. OECD, 2025). Since this country-level mediation is the primary focus of the present study, we review cross-national research in the following section, rather than student-level analyses.

## Review of country-level research on ECEC participation rates, socioeconomic inequalities in participation, and socioeconomic inequalities in school achievement

A few studies have empirically tested whether increases in ECEC participation rates lead to reductions in socioeconomic inequalities in ECEC participation at the country level. While cross-sectional studies report that countries with higher participation rates tend to exhibit somewhat lower levels of socioeconomic inequality in participation (Van Lancker, 2013; Van Lancker & Ghysels, 2016), longitudinal analyses provide more mixed findings. A study of OECD countries found that increases in ECEC enrollment rates do not universally correspond to decreases in participation gaps; in many countries, these gaps persist, and in some cases, they even widen (OECD, 2025). Similarly, Yang and colleagues (2024) found that among 20 countries with rising ECEC enrollment rates, only six showed a significant reduction in enrollment inequalities, while 13 did not exhibit a substantial change. The literature discusses different explanations for these mixed findings. When government involvement is weak and ECEC provision is largely market-based, newly available ECEC services tend to disproportionately benefit more affluent families; when governments actively subsidize or provide ECEC services, by contrast, more equitable access is generally expected (Van Lancker & Ghysels, 2016; Yang et al., 2024).

Three studies investigated associations between countries’ ECEC enrollment rates and achievement scores of socioeconomically disadvantaged students or socioeconomic inequalities in achievement scores, respectively. One study used data from multiple cycles of two international large-scale assessment studies, the Progress in International Reading Literacy Study (PIRLS) and the Programme for International Student Assessment (PISA) (Strietholt et al., 2020). In the main analyses, they investigated if within-country trends in preschool enrollment rates affected countries’ average achievement scores in the PIRLS and PISA tests and did not find significant effects. They repeated the analyses for subsamples of socioeconomically advantaged and disadvantaged students and found no significant effects, either. However, this study did not consider whether increases in enrollment rates were associated with decreases in socioeconomic inequalities in enrollment rates in the countries of interest. A second study used data from PISA 2006 and investigated the socioeconomic inequalities in PISA test scores between countries with preschool enrollment rates above and below 75% (Schlicht et al., 2010). They found more pronounced degrees of socioeconomic inequality in countries with low preschool enrollment rates. Similarly, a third study used data from the Trends in International Mathematics and Science Study (TIMSS) in 1995 and 1999 and investigated the association between countries’ pre-primary enrollment rates and the role the family background plays for test scores (Schütz et al., 2008). They found a non-linear relationship that they interpreted as follows: “Educational opportunities get more unequal with rising pre-school enrollment up to a maximum of 61 percent of pre-school enrollment. Only beyond this threshold is higher pre-school enrollment associated with more equal educational opportunities” (ibid., p. 301). Furthermore, they found that a longer duration of preschool education was associated with lower inequalities in TIMSS test scores.

The present study builds on this body of research by directly testing the mediation hypothesis that is only implicitly suggested by previous work: namely, that increasing ECEC participation rates can reduce socioeconomic inequalities in educational achievement—especially if such increases are accompanied by a narrowing of participation gaps between socioeconomic groups. To the best of our knowledge, this is the first study to empirically test this mediation mechanism.

### The present study

This study addresses two primary research questions:

1. What are the international trends in (a) ECE participation rates, (b) socioeconomic inequalities in ECE participation, and (c) socioeconomic inequalities in reading achievement at the end of primary school?
2. Do increasing ECE participation rates lead to reductions in socioeconomic inequalities in ECE participation, and thereby to decreases in socioeconomic inequalities in reading achievement?

**Fig. 1:**
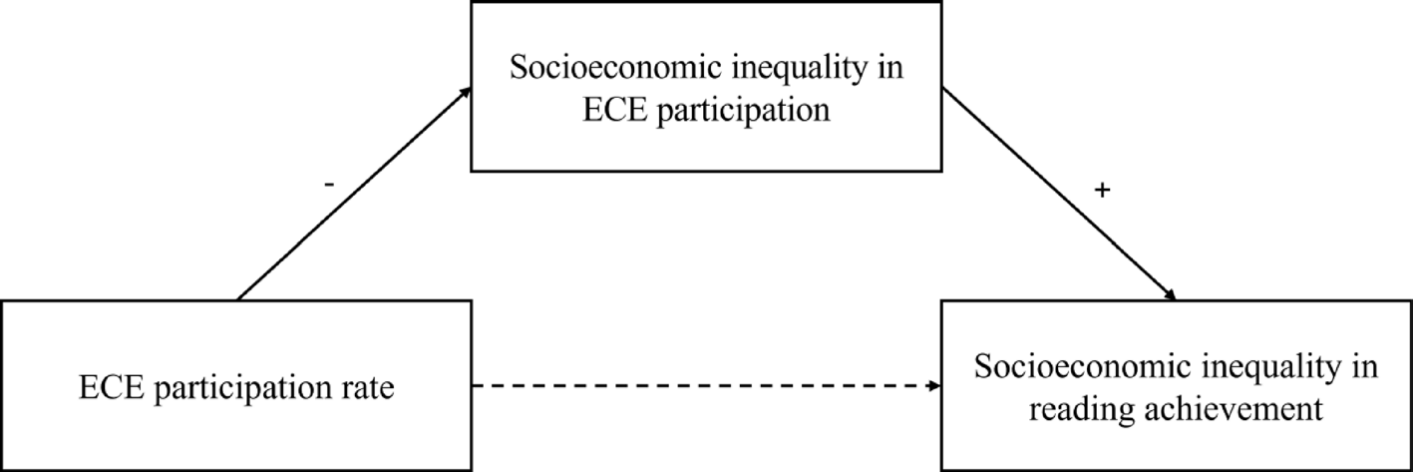
Schematic representation of main research question

To investigate these questions, we conduct country-level trend analyses using country-fixed effects models. To answer the first research question, we perform three separate trend analyses—one for each variable of interest. To address the second and main research question, we estimate a mediation model (see schematic display in Fig. 1), which tests the hypothesis that the association between increasing ECE participation rates and decreasing socioeconomic inequalities in reading achievement is fully mediated by reductions in socioeconomic inequalities in ECE participation. Assumptions that have to be met to interpret this mediation causally are discussed.

## Methods

### Data

This study analyzes data from the international large-scale assessment PIRLS (Mullis et al., 2023; https://www.iea.nl/data-tools/repository/pirls). Specifically, we include data from all countries that participated in at least two of the five PIRLS cycles (2001, 2006, 2011, 2016, 2021^2^); in total 225 country-by-year observations from 64 education systems.^3^ To account for countries’ economic and demographic developments over time, we supplement the PIRLS data with external indicators.

**Fig. 2:**
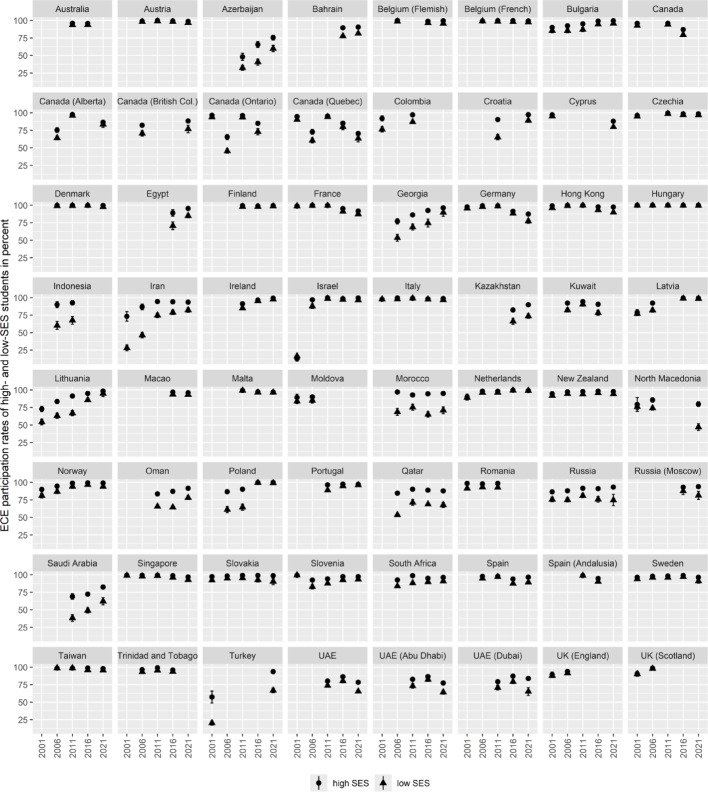
ECE participation rates of high- and low-SES students in percent by country and year. *Note* Displayed are ECE participation rates of high-SES (points) and low-SES (triangles) students ± 1.96 *SE (vertical lines)

**Fig. 3:**
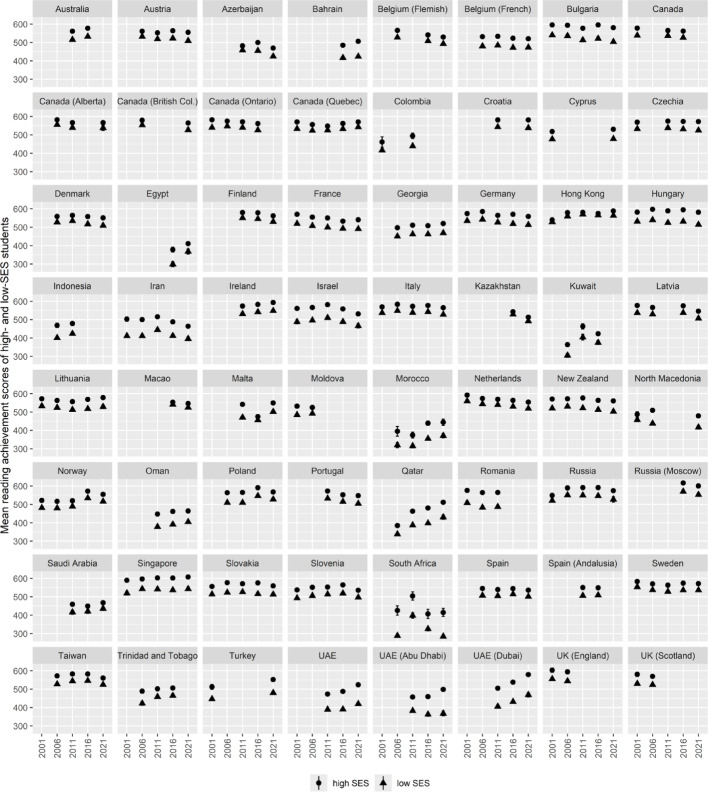
Mean reading achievement of high- and low-SES students by country and year. *Note* Displayed are mean reading achievement scores of high-SES (points) and low-SES (triangles) students ± 1.96 *SE (vertical lines)

Figures 2 and 3 provide an overview of the countries and years included in the analysis. PIRLS is selected for this study because it is an international large-scale assessment that provides both achievement test scores and student background data—including information on ECE participation and socioeconomic status (SES). Since country-fixed effects analysis relies on repeated measurements over time, we furthermore needed an assessment with multiple cycles and comparable measures over time.^4^ PIRLS is a comprehensive assessment of students’ reading achievement and learning conditions at the end of ISCED Level 1 (i.e., primary education) and is administered internationally to students in grade 4^5^ (Mullis et al., 2023). The assessment combines standardized reading tests with questionnaire data collected from students, their parents or legal guardians, teachers, school leaders, and national education authorities. To ensure nationally representative samples, PIRLS employs a two-stage sampling design: first selecting schools, then randomly sampling intact classes within those schools.

### Variables

#### ECE participation rates

Mean ECE participation rates by country and year were computed using data from the PIRLS home questionnaires. In the 2001, 2006, and 2011 cycles, parents or legal guardians were asked, “Did your child attend <ISCED Level 0>?” with response options of 1 = *yes* and 2 = *no*. If they answered yes, a follow-up question asked, “How long was he/she in <ISCED Level 0>?” (with five response options; see Appendix 1 for details). In these questions, the placeholder “<ISCED Level 0>” was replaced with country-specific terminology for ECE. In the 2016 and 2021 cycles, the home questionnaire included three items instead of two, distinguishing between ISCED Level 0 programs for children under the age of three and those for children aged three and older (see Appendix 1). For consistency across survey cycles, we recoded these items to create a unified binary indicator of general ECE participation (0 = *no*, 1 = *yes*) for the main analyses.

Using this binary variable, we calculated weighted mean ECE participation rates for each country-by-year observation. We employed the ‘EdSurvey’ package (Bailey et al., 2017) in the R environment (R Core Team, 2025), which accounts for PIRLS’ complex sampling design. Country-by-year observations that did not administer the ECE participation items (e.g., the United States) were excluded from the analyses.

Across the 225 country-by-year observations, mean ECE participation rates ranged from 17.8% to 99.9% (*M* = 88.1, SD = 13.8). Although parent-reported ECE participation data from representative samples of primary school children offer a valid and reliable basis for estimating national participation rates, this measure is subject to missing data concerns. The proportion of missing responses varied widely, from 0.4% to 67.3% across observations. However, the PIRLS-based participation trends closely align with an external benchmark: UNESCO’s gross enrollment ratio in pre-primary education (‘SE.PRE.ENRR’ indicator; https://databank.worldbank.org/metadataglossary/world-development-indicators/series/SE.PRE.ENRR) that is based on administrative data (*r* = .887 in country-fixed effects models). We consider *r* = .887 a high degree of alignment, also because the UNESCO enrollment ratio is distorted by children who are enrolled but too young or old given the target age of ECE in the country (i.e. the gross ratio can exceed 100%).

#### Inequality in ECE participation between low- and high-SES children

To assess socioeconomic inequality in ECE participation, we calculated the difference in participation rates between children from low- and high-SES backgrounds for each country-by-year observation. SES was operationalized based on parental education, using data from the PIRLS home questionnaires. Specifically, students were classified as low-SES if neither parent or guardian had attained at least ISCED Level 5 (short-cycle tertiary education), and as high-SES if at least one parent or guardian had completed ISCED Level 5 or higher. The home questionnaires varied in response categories across PIRLS cycles (see Appendix 1), but it was possible to harmonize responses into below ISCED Level 5 and ISCED Level 5 or above. If valid data about two parents/guardians were available, we used the highest. If valid data was available for only one parent/guardian, we used this valid answer, and if no valid data was available (including the ‘not applicable’ category), the parental education variable was missing. Again, we computed the gaps in ECE participation rates between high- and low-SES students with the ‘EdSurvey’ package (Bailey et al., 2017) in the R environment (R Core Team, 2025).

Figure 2 displays the ECE participation rates of high- and low-SES students. Across the country-by-year observations, the participation gaps ranged from − 3.6 to 45.0 percentage points (M = 7.6, SD = 8.7). Positive values indicate higher participation rates among high-SES students. The proportion of missing data on parental education varied substantially, from 1.6% to 68.0% across observations. Despite this, the PIRLS-based SES indicator aligned closely with an external benchmark—the UNESCO-reported indicator (‘SE.TER.CUAT.ST.ZS’ indicator; https://databank.worldbank.org/metadataglossary/world-development-indicators/series/SE.TER.CUAT.ST.ZS) of the percentage of adults (aged 25+) who have completed at least short-cycle tertiary education (*r* = .928 in country-fixed effects models).

#### Inequality in reading achievement between low- and high-SES students

We computed the socioeconomic inequality in reading achievement based on the SES variable (see above) and the overall reading comprehension scale. The PIRLS reading comprehension test consists of multiple reading passages and accompanying test items, assessing both reading to acquire and use information and reading for literary experience. To minimize the test burden, each student responds to two booklets that contain only parts of the test. PIRLS provides five plausible value estimates for reading achievement per student to reflect measurement error in the test scores. Using the ‘EdSurvey’ package (Bailey et al., 2017) in the R environment (R Core Team, 2025), we computed the unstandardized mean difference in reading achievement between low- and high-SES students per country-by-year observation, while taking the complex sampling design in PIRLS into account and handling the plausible values according to Rubin’s (1987) rules.

Figure 3 presents the mean reading scores for high- and low-SES students. Across the 225 country-by-year observations, the SES-based achievement gap ranged from 9.56 to 137.14 points (M = 49.57, SD = 21.20). Positive values indicate higher average reading scores among high-SES students. As noted earlier, missing data were present for the SES variable, but there was no missing data in the reading achievement plausible values.

#### Control variables

The aim of the second research question is to trace back trends in inequalities in reading achievement between low- and high-SES children to trends in ECE participation rates and socioeconomic inequalities in ECE participation rates. We included a set of control variables to be able to account for further major economic, demographic, or policy changes that might be associated with ECE participation trends and thus bias the associations of interest.

We first included Gross Domestic Product (GDP) per capita, as reported by the World Bank (‘NY.GDP.PCAP.KD’ indicator; https://data.worldbank.org/indicator/NY.GDP.PCAP.KD) to be able to control for major changes in countries’ ability to allocate funding to education. Economic development may influence both ECE participation rates and broader structural features of education systems that impact socioeconomic inequality. For countries with multiple education systems (e.g., Belgium Flemish and Belgium French), we assigned the same national GDP value. The GDP variable was available for all country-by-year observations except Taiwan (n = 4 missing country-by-year observations). Across observations, GDP per capita ranged from $1,432 to $94,184 (M = $29,105, SD = $19,315), measured in constant 2015 U.S. dollars. We aimed to include an indicator for income inequality, since we assumed that changes in parents’ disposable income can affect both socioeconomic inequalities in ECE participation and later school achievement. However, the available indicators (e.g., from the World Bank) covered only about half of our country-by-year observations, which is why we decided not to include them as control variables.

Second and third, we included two demographic controls: the size of the population of children aged 0–14, from the World Bank (‘SP.POP.0014.TO’ indicator; https://data.worldbank.org/indicator/SP.POP.0014.TO), and the net migration (i.e., the number of immigrants minus the number of emigrants, including both citizens and noncitizens), also from the World Bank (‘SM.POP.NETM’ indicator; https://data.worldbank.org/indicator/SM.POP.NETM). Major changes in the number of children and of immigrants might be associated with ECE participation rates as well as socioeconomic inequalities in ECE participation. Like above, we assigned education systems from the same country the same population size values. Both the number of children and net migration variables are available for all observations except Taiwan (n = 4 missing country-by-year observations). The number of children per observation ranged from 60,460 to 68 million (M = 5.4 million, SD = 8.8 million), and net migration from -879,235 to 659,720 (M = 76,207, SD = 159,151).

Fourth, it might be that ECE expansion policies coincide with other educational reforms that might affect changes in socioeconomic inequalities in reading achievement at the end of primary school. In this case, our country-level trend analyses on effects of ECE participation would be biased by effects of these other policies. Since there is no systematic overview of such educational reforms in the primary school sector across the education systems under investigation, we cannot include a direct control variable for such policies. We do however believe that horizontal differentiation policies that affect the degree to which high- and low-SES students attend the same schools (e.g., school-choice regulations, private schooling) have the potential to affect socioeconomic achievement inequalities, because they create the possibility that high- and low-SES students experience different learning conditions at school (e.g., Burger, 2019; Strello et al., 2022; Strietholt et al., 2019). We therefore included the degree of between-school segregation of high- and low-SES students as control variable. Specifically, we computed the dissimilarity index (Duncan & Duncan, 1955), which can range between 0 (i.e., all schools in a country-by-year observation have the same proportion of high-SES children) and 1 (i.e., no school has both high- and low-SES students). Across country-by-year observations, the dissimilarity index ranged between 0.22 and 0.63 (M = 0.40, SD = 0.08).

In a similar vein, we assume that vertical differentiation policies, such as school entry or grade retention policies, have the potential to affect changes in socioeconomic achievement inequalities in PIRLS, if they imply that high- and low-SES students vary in age when they participate in the assessment. If such a policy would mean that low-SES students entered school later, on average, or repeated a grade more often than high-SES students (e.g., González-Betancor & López-Puig, 2016; Janus & Duku, 2007), PIRLS would compare older, more mature low-SES students with younger, less mature high-SES students, which might reduce measured socioeconomic inequalities in achievement. We therefore computed the mean age difference between high- and low-SES students for each country-by-year observation and included it as a fifth control variable. Across country-by-year observations, the mean age gap ranged between − 0.73 and 0.08 (M = − 0.09, SD = 0.11).

### Analyses

#### Analyses for first research question on international trends

The first research question examines international trends in (a) ECE participation rates, (b) socioeconomic inequalities in ECE participation, and (c) socioeconomic inequalities in reading achievement. To estimate these trends, we leveraged the repeated participation of countries across PIRLS cycles (see Figs. 2 and 3). Specifically, we employed country-fixed effects models, regressing each of the three outcome variables—ECE participation rates, the SES-based gap in ECE participation, and the SES-based gap in reading achievement—on PIRLS cycle indicators and country identifiers in separate models. This fixed effects approach isolates within-country variation over time, thereby controlling for all time-invariant differences between countries. Consequently, the estimated PIRLS cycle coefficients represent international trends in these variables, unaffected by the unbalanced participation of countries across PIRLS cycles.

#### Analyses for second research question on mediation effects

To address the second and main research question concerning the potential mediating role of socioeconomic inequalities in ECE participation on the relationship between overall ECE participation rates and socioeconomic inequalities in reading achievement, we first estimated three separate country-fixed effects regression models corresponding to the individual paths in the mediation model. Subsequently, we estimated the full mediation model (see Fig. 1). The mediation analysis was conducted using the ‘lavaan’ package (Rosseel, 2012) in R (R Core Team, 2025). These country-fixed effects regression models effectively compare countries with themselves over time, accounting for international trends and all time-invariant between-country differences.

In the separate mediation path models, we also included control variables—GDP per capita, population size of children, net migration, between-school segregation of high- and low-SES students, and the mean age gap between high- and low-SES students—to account for major economic, demographic, and policy changes within countries over time (see control variables’ section above).

#### Prerequisites of country-fixed effects regression

Estimating fixed effects models requires certain conditions to be met (e.g., Allison, 2009). First, country-fixed effects trend analyses need longitudinal data with comparable measurements over time. PIRLS is designed to facilitate such country-level trend analyses by providing linkable test scores, comparable questionnaires, and consistent sampling procedures; Von Davier et al., 2023). While the comparability of reading scores is well established (ibid.), we ensured that the cycle-specific ECE participation and SES variables were recoded to maximize comparability (see variables’ section and Appendix 1).

Second, fixed-effects regression requires sufficient within-country variation in predictor variables (Allison, 2009). Although some countries exhibit stable, high ECE participation rates and low inequalities in ECE participation between socioeconomic groups (see Fig. 2), most countries show meaningful variation over time.

Third, the method assumes no reciprocal or reverse causation (ibid.); that is, socioeconomic inequalities in ECE participation or reading achievement should not causally influence overall ECE participation rates, and inequalities in reading achievement should not influence inequalities in ECE participation. In the present case, we assume that countries that experience *increases* in socioeconomic inequalities in either ECE participation or reading achievement would strive to *increase* ECE participation and *decrease* inequalities in ECE participation. Thus, reverse causality issues seem unlikely alternative explanations for the expected, opposite relationships in the present study (see Fig. 1).

Fourth, fixed-effects regression assumes that time-invariant predictors have constant effects on the outcomes across time (ibid.)—in this study, that ECE participation rates have consistent effects on inequalities in participation, and that inequalities in participation have consistent effects on inequalities in reading achievement. This assumption is not testable in the present study.

Finally, it is crucial to control for time-varying confounders that might provide alternative explanations for observed associations (ibid.). Therefore, in our analyses addressing the second research question, we adjust for major economic, demographic, and policy changes that could affect both ECE participation rates and inequalities in participation and educational outcomes. Additional potential control variables that we were unable to include are discussed in the study’s limitations section.

## Results

### Results of the trend analyses (research question 1)

**Fig. 4:**
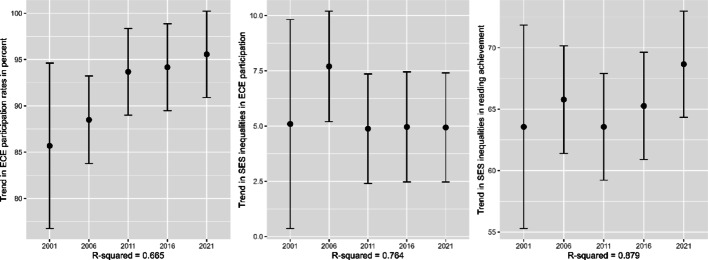
Trends in ECE participation rates, socioeconomic inequalities in ECE participation rates, and socioeconomic inequalities in reading achievement (research question 1). *Note* Displayed are the results of three separate country-fixed effects trend analyses ± 1.96 *SE (vertical lines). The SEs for 2001 are large because they reflect the SEs in Bulgaria in 2001, while the SEs of the other cycles reflect international results. See Appendix 2 for the numerical model results

The first country-fixed effects trend analysis revealed a significant international increase in ECE participation rates from 2001 to 2021 across participating countries (see left panel of Fig. 4). Specifically, participation rates in 2011, 2016, and 2021 were significantly higher than those in 2001. However, the second analysis provided no evidence of an international change in the ECE participation gap between high- and low-SES students; only the 2006 gap exceeded the 2001 value significantly (see middle panel of Fig. 4). The third analysis found no significant international trend in socioeconomic inequality in reading achievement; only the 2021 measure exceeded the one from 2001 significantly (see right panel of Fig. 4).

As reflected in the models’ R^2^ values (Fig. 4), these international trends and stable between-country differences explained a large proportion of the variation in country-by-year observations, while the remaining unexplained variance reflects country-specific trends over time.

### Results of the mediation analyses (research question 2)

**Fig. 5:**
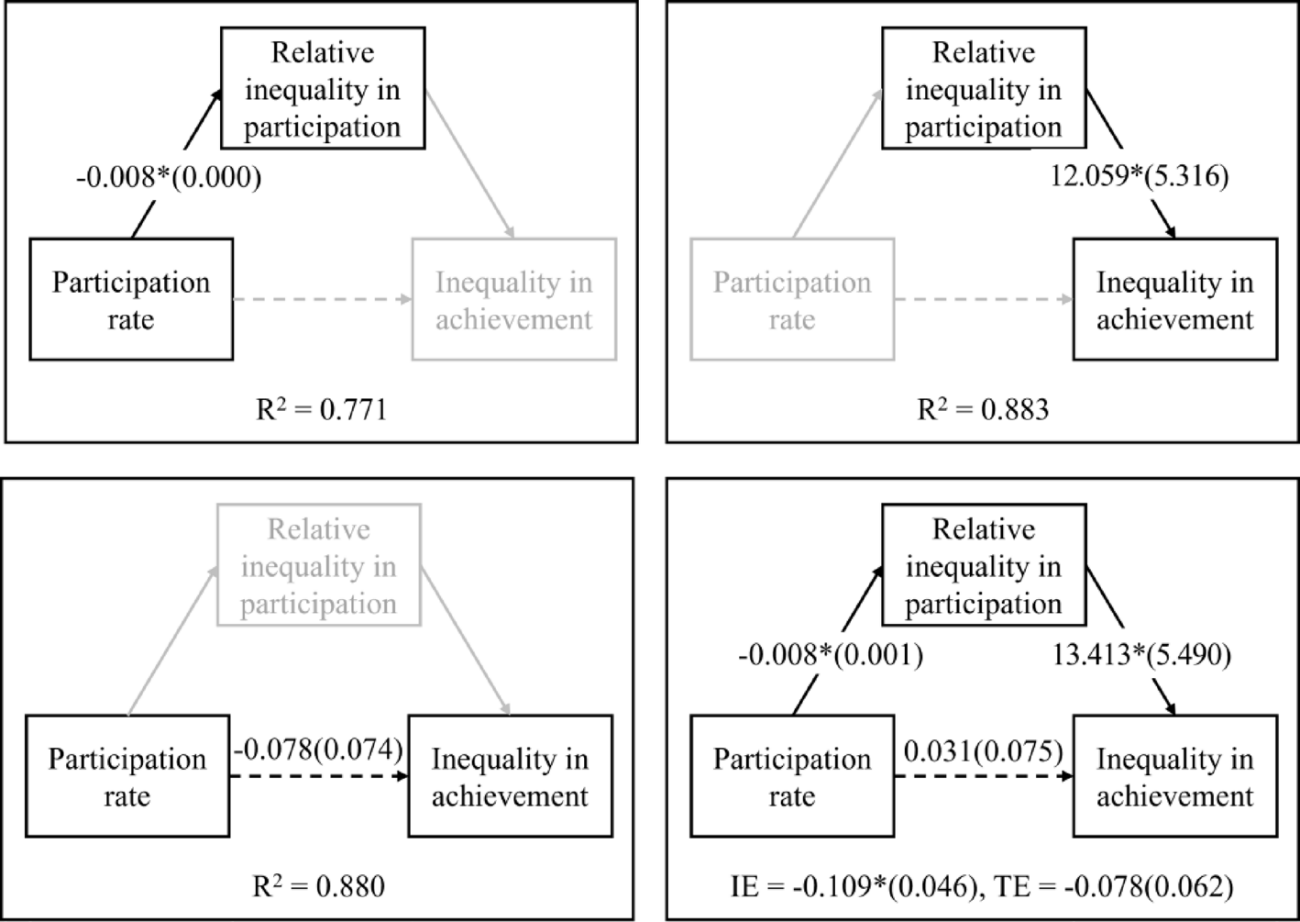
Results of the single paths and full mediation analyses (research question 2). *Note* Displayed are unstandardized coefficients from three separate country-fixed effects regression analyses and a country-fixed effects mediation model. Variables and paths that are printed grey were not included in the models. IE = indirect effect, TE = total effect. *p < .05

We first estimated the individual paths of the mediation model using separate country-fixed effects regression models (see top-left, top-right, and bottom-left panels in Fig. 5). As hypothesized, we found a significant negative association between overall ECE participation rates and socioeconomic inequalities in ECE participation (top-left panel), and a significant positive association between socioeconomic inequalities in ECE participation and inequalities in reading achievement (top-right panel). The ECE participation rates did not significantly predict reading achievement inequalities (bottom-left panel). These findings remained consistent after including the five control variables—GDP per capita, number of children, net migration, between-school segregation, and mean age difference between SES groups (see Appendix 3).

Next, we estimated the full mediation model (bottom-right panel, Fig. 5). The results supported our hypothesized mediation model (Fig. 1), showing that increasing ECE participation rates were indirectly associated with a small but significant decrease in socioeconomic inequalities in reading achievement, when they were associated with reduced inequalities in ECE participation (i.e., significant, negative indirect effect). Due to the complexity of the model and limited number of observations, we were unable to include the control variables in this full mediation analysis.

### Further analyses on longer ECE participation

In the main analyses, we examined general ECE participation (0 = *no*, 1 = *yes*), noting rates close to 100% in many country-by-year observations (see Fig. 2). To extend these findings, we conducted additional analyses focusing on longer ECE participation (0 = *no participation or participation for less than two years*, 1 = *participation for at least two years*; see Appendix 1 for item wording across PIRLS cycles). All main analyses were repeated using this longer participation variable.

Across country-by-year observations, the rate of students with a longer ECE participation varied between 4.9% and 98.6% (M = 71.3, SD = 23.2), with missing data rates ranging from 1.1% to 67.3%. Equivalent to the general participation variable, we computed socioeconomic inequalities in longer ECE participation as the mean difference in longer participation rates between high- and low-SES students per country-by-year observation. This measure ranged from -3.9 to 40.5 percentage points (M = 11.2, SD = 8.9), indicating that high-SES students were generally overrepresented among longer-term participants (see Fig. 7 in Appendix 4).

The country-fixed effects trend analyses revealed a marked international increase in longer ECE participation between 2001 and 2021, but no evidence of a decrease in socioeconomic inequality in longer participation (see Fig. 8 in Appendix 4). As in the main analyses, mediation path analyses showed a significant negative relationship between longer ECE participation rates and socioeconomic inequalities in longer participation (top-left panel, Fig. 9) and no significant relationship between longer ECE participation rates and reading achievement inequality (bottom-left panel). Unlike in the main analyses, however, the path from inequalities in longer participation to inequalities in reading achievement was not significant (top-right panel). These results remained consistent after including control variables. In the full mediation model, the indirect effect of longer ECE participation rates on reading achievement inequality through socioeconomic inequalities in longer ECE participation failed statistical significance, even though the path between inequality in longer participation and inequality in achievement was significant in this model (bottom-right panel).

### Further analyses on relative inequality in ECE participation

In the main analyses, we used an absolute measure of inequality in ECE participation. These absolute differences in participation are, however, constrained by the overall participation rate: in countries with participation rates close to 100% (see Fig. 2), there is limited scope for large absolute SES gaps. We therefore repeated the main analyses using a relative measure. To calculate the relative inequality measure, we divided the absolute SES gap in participation by the overall participation rate. This resulting metric reflects the extent of the socioeconomic inequality in participation in relation to the general level of ECE participation in a country. The relative SES inequality measure ranged from -0.20 to 1.53 across country-by-year observations (M = 0.11, SD = 0.18).

**Fig. 6:**
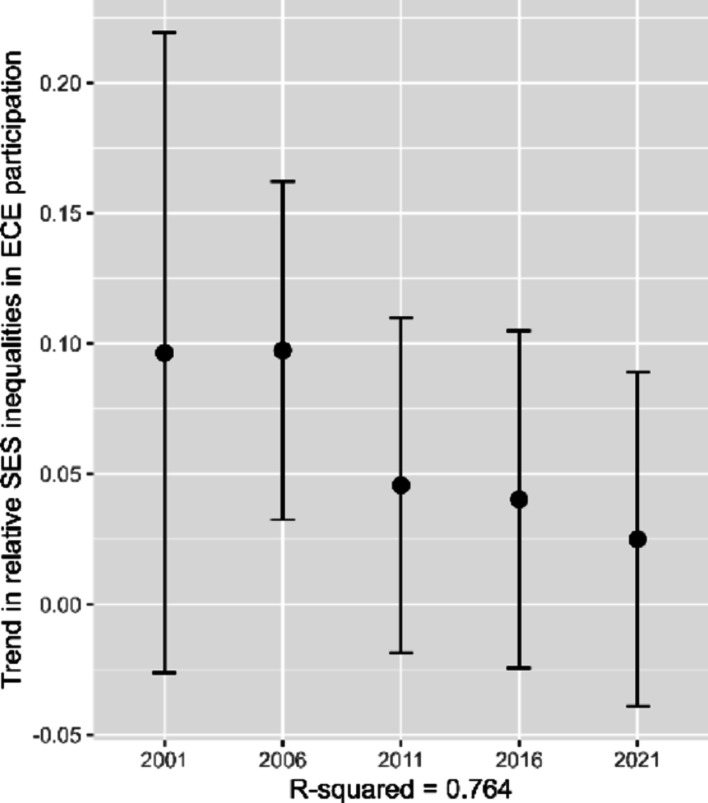
Trends in relative socioeconomic inequalities in ECE participation rates. *Note* Displayed are the results of a country-fixed effects trend analyses ± 1.96 *SE (vertical lines). The SEs for 2001 are large because they reflect the SEs in Bulgaria in 2001, while the SEs of the other cycles reflect international results

**Fig. 7:**
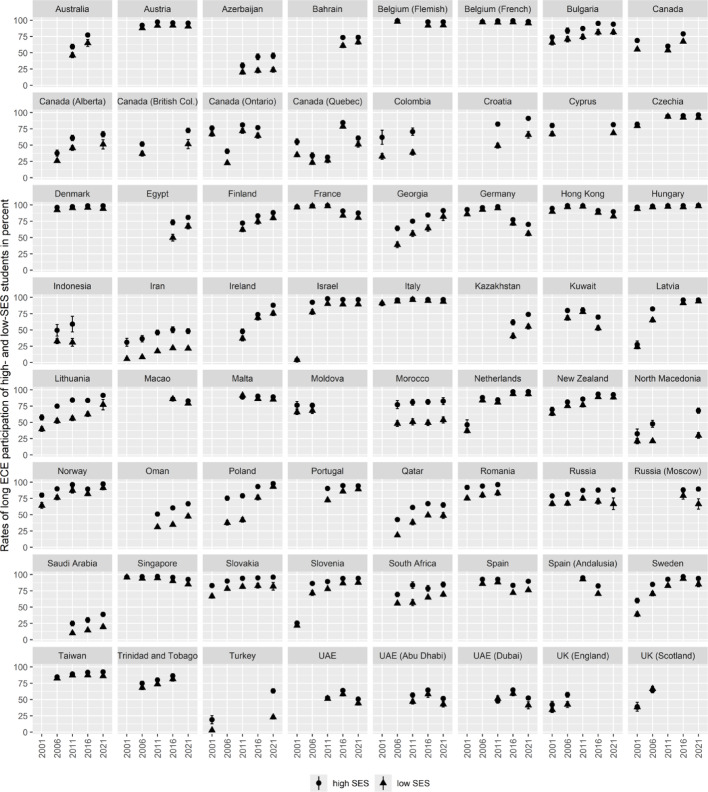
Longer ECE participation rates of high- and low-SES students in percent by country and year. *Note* Displayed are longer ECE participation rates (0 = *no participation or participation for less than two years*, 1 = *participation for at least two years)* of high-SES (points) and low-SES (triangles) students ± 1.96 *SE (vertical lines)

**Fig. 8:**
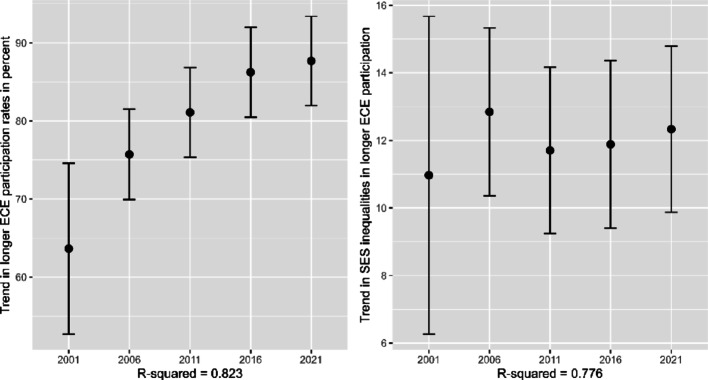
Trends in longer ECE participation rates and socioeconomic inequalities in longer ECE participation rates. *Note* Displayed are the results of two separate country-fixed effects trend analyses ± 1.96 *SE (vertical lines). The SEs for 2001 are large because they reflect the SEs in Bulgaria in 2001, while the SEs of the other cycles reflect international results

**Fig. 9:**
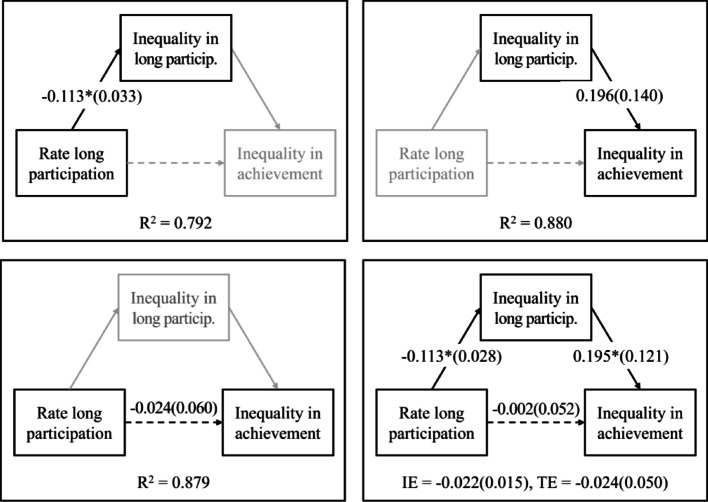
Results of the single paths and full mediation analyses in further analyses on longer ECE participation. *Note* Displayed are unstandardized coefficients from three separate country-fixed effects regression analyses and a country-fixed effects mediation model. Variables and paths that are printed in grey were not included in the models. IE = indirect effect, TE = total effect. *p < .05

The country-fixed effects trend analysis indicated a trend towards declining relative inequality in ECE participation, with the 2021 measure being significantly smaller than the one from 2001 (see Fig. 6). The results of the separate and full mediation results replicated the main findings (see Appendix 5). We found a significant negative association between ECE participation rates and relative socioeconomic inequalities in ECE participation (top-left panel), a significant positive association between relative socioeconomic inequalities in ECE participation and inequalities in reading achievement (top-right panel), and a non-significant association between ECE participation rates and reading achievement inequality (bottom-left panel). These findings remained consistent after including the control variables. The results of the full mediation model (bottom-right panel) showed a small but significant indirect negative effect of ECE participation rates on socioeconomic inequalities in reading achievement through relative inequalities in ECE participation.

### Robustness checks

We conducted robustness checks to ensure our results were not driven by data quality limitations. Since countries participating in only two PIRLS cycles provided a limited data basis for country-fixed effects analyses, we repeated the main analyses using a subset of countries with participation in at least three cycles (n = 193 country-by-year observations). The trend, mediation path, and full mediation analyses mirrored the main findings.

Given the high proportion of missing data in the ECE participation and SES variables from home questionnaires in some country-by-year observations, we also performed robustness checks restricting the sample to cases with at most 30% missing data on these variables (n = 182 country-by-year observations). These analyses largely produced results consistent with the main trend and mediation analyses. However, both the increase in inequality in ECE participation in 2006 and the increase in inequality in achievement in 2021 that were observed in the main trend analyses, were not significant in this subset of observations.

## Discussion

### International trends over time

Consistent with previous reports (European Commission: Directorate-General for Education, Youth, Sport and Culture, 2024; Eurostat, 2024; OECD, 2025; OECD Family Database, 2024), our analysis of PIRLS data revealed an international increase in ECE participation rates over time. Across the included country-by-year observations, the international, overall ECE participation rate, as reported by parents or legal guardians, rose from 86% in 2001 to 96% in 2021. Levels and trends varied between countries: many highly developed Western countries have maintained consistently high participation rates for a long time, while others exhibited clear increases or even declines in recent years. When examining longer ECE participation (defined as at least two years), we found similar patterns but with generally more pronounced differences between countries. Internationally, longer participation rates increased from 64% in 2001 to 88% in 2021.

Also consistent with previous literature (e.g., Hogrebe & Strietholt, 2016; Kulic et al., 2019; OECD, 2025; Van Lancker, 2013; Waldfogel et al., 2023), we found that socioeconomically privileged children, here measured by tertiary parental education, had either equally high or higher rates of ECE participation than less privileged children in almost all country-by-year observations. Consistent with previous research (OECD, 2025; Yang et al., 2024), we did not find evidence for an international increase or decline in the absolute socioeconomic inequality in ECE participation across countries (except an increase in 2006 that was not significant in a robustness check with fewer observations). Further analyses that used a relative measure (i.e., inequality in ECE participation relative to the general level of ECE participation), however, provided some evidence for an international decline in the relative socioeconomic inequality in ECE participation between 2001 and 2021. In light of the fact that in countries with participation rates close to 100%, the absolute difference in participation rates of high- and low-SES students cannot exceed a few percentage points, our findings using a relative measures for participation inequalities indicate a promising avenue for future research that could have a closer look at implications of different operationalizations of inequalities in ECE participation.

Unsurprisingly in light of vast amounts of research (e.g., Korous et al., 2022; Liu et al., 2022), we found that high-SES students performed better on the PIRLS reading tests than low-SES students across country-by-year observations. We did, however, not find a clear, international trend across time points, except an increase in 2021 (which was not significant in a robustness check using fewer observations). In light of previous research, educational disruptions due to the Covid pandemic that exacerbated educational inequalities might explain this increase in 2021 (Jakubowski et al., 2025; Kennedy & Strietholt, 2023).

### Link between participation rates, inequalities in participation, and inequalities in reading achievement

The primary research question of this study examined whether increases in ECE participation rates lead to reductions in socioeconomic inequalities in ECE participation, and consequently to decreases in socioeconomic inequalities in reading achievement. The findings of our mediation path and full mediation analyses supported this hypothesized relationship, while certain methodological limitations have to be acknowledged.

Higher ECE participation rates were associated with lower relative socioeconomic inequalities in ECE participation. Specifically, a one percentage point increase in overall ECE participation was associated with approximately one third of a percentage point decrease in the socioeconomic inequality in ECE participation in the main mediation path analysis. Similar results were observed in analyses with control variables, in further analyses, and in robustness checks. Previous research reported weaker or less consistent associations between countries’ rising enrollment rates and socioeconomic inequalities in enrollment using different datasets (OECD, 2025; Yang et al., 2024).

Furthermore, we found that countries experiencing reductions in inequalities in ECE participation also showed decreases in inequalities in reading achievement. According to the main mediation path model, a one percentage point decrease in socioeconomic inequalities in ECE participation was associated with a one-third of a score point decrease in socioeconomic inequalities in reading achievement (mean socioeconomic inequality in reading achievement was about 50 score points across country-by-year observations). Again, results of analyses with control variables, of further analyses, and robustness checks mirrored this finding, except the further analysis on longer preschool participation. To our knowledge, this is the first study to investigate this association empirically.

In the full mediation model, these relationships formed a significant negative indirect effect of ECE participation rates on inequalities in achievement through inequalities in participation rates. The magnitude of this indirect effect was small. Specifically, a one percentage point increase in ECE participation was linked to a 0.14 score point decrease in socioeconomic inequalities in reading achievement at the country level—an outcome influenced by many factors beyond ECE participation and difficult to modify through policy reforms. The direct effect of participation rates on reading achievement inequalities was not statistically significant in the full mediation model, and the path from ECE participation rates to inequalities in achievement was not significant in the separate mediation path model. These findings have to be interpreted in light of certain limitations.

### Limitations

We would like to highlight several key limitations of the present study. Our use of country-fixed effects models allows for comparison of countries with themselves over time, thereby controlling for time-invariant between-country differences. While this longitudinal design enhances the potential for causal inferences (Allison, 2009), it remains crucial to consider and account for possible time-variant confounders. For instance, if countries with rising ECE participation systematically differ from those with stable or declining rates in other ways, these unmeasured factors could offer alternative explanations for the observed associations. To mitigate this, we included control variables capturing major economic (GDP per capita), demographic (population size of children and net migration), and policy-related changes (proxies for horizontal and vertical differentiation). Nevertheless, we cannot fully exclude the possibility that ECE expansion policies coincided with other reforms not captured in our controls—such as the introduction of remedial education programs for low-performing students or increased teacher-to-student ratios—both of which could independently influence socioeconomic inequalities in academic achievement. Future research should strive to incorporate even more comprehensive measures of policy changes. Similarly, including additional economic controls such as income inequality would be valuable, although data limitations prevented their inclusion here without significant loss of statistical power.

Similarly, the causal interpretability of the mediation model is limited by the fact that model complexity prevented us from including the control variables in the full model. However, the path coefficients resembled the ones from the separate path models that included controls. A more general challenge in the mediation model is to ensure that decreases in socioeconomic inequalities in ECE participation actually go back to increases in overall participation rates. This assumption rests on the fact that this is a designated goal of many participation expansion policies (e.g., European Commission: Directorate-General for Education, Youth, Sport and Culture, 2024) and that participation rates are already very high among socioeconomically privileged groups in many countries (e.g., OECD, 2025).

Although PIRLS data are well-suited for addressing our research questions (see footnote 4), some limitations persist. The wording of key variables—ECE participation and SES—varied somewhat across cycles, and both variables suffered from missing data. We conducted data quality checks by comparing country-level trends in these variables with external UNESCO measures, finding strong alignment. UNESCO data do not permit measurement of socioeconomic inequalities in ECE participation. Additionally, we ran robustness checks excluding countries with limited PIRLS participation or high missingness, which largely confirmed our results.

With the PIRLS data, it is not possible to consider the exact duration of ECE participation across cycles (beyond the further analyses in this study), or the quantity of ECE participation per week (for which it is difficult to find internationally comparative data; see OECD Family Database, 2024). Similarly, no information is available on the quality of the ECE programs, which would be especially interesting since low-SES students often experience lower-quality ECE than high-SES students (OECD, 2025). Accounting for quality differences could potentially reveal stronger associations between participation rates, inequalities in participation, and inequalities in achievement than those observed in our study.

Finally, despite using data from all five PIRLS cycles and including countries with at least two participations, our sample of 225 country-by-year observations across 64 education systems remains modest. This limited sample size likely reduced statistical power, which may explain why some robustness checks with smaller samples did not reach significance. Future PIRLS cycles will incrementally increase the available data, enhancing the power of country-level trend analyses such as ours.

## Conclusions

Despite these limitations, our findings contribute to prior research on the link between ECE participation rates and socioeconomic inequalities in academic achievement at the country level (Schlicht et al., 2010; Schütz et al., 2008). While these few previous studies investigated direct associations between overall ECE participation rates and achievement inequality outcomes (a direct association that was not significant in our study), the contribution of the present study is to explicitly model the indirect role that inequalities in ECE participation play in this relationship (an indirect association that was significant in our study).

Given that some untestable assumptions hold (see section on prerequisites of country-fixed effects regression), we interpret our findings as follows: increases in countries’ overall ECE participation rates seem to contribute to reductions in socioeconomic inequalities in reading achievement at the end of primary school, when accompanied by decreases in socioeconomic disparities in ECE participation. This finding offers an encouraging signal for countries currently investing in, or planning to expand, universal ECE. Our results underscore that ECE expansion policies should explicitly target socioeconomic inequalities in participation in order to effectively reduce later educational disparities. This emphasis is particularly important given prior research demonstrating that socioeconomically disadvantaged children often benefit more from ECE participation than their more privileged peers (Schmutz, 2024; Van Huizen & Plantenga, 2018).

## Figures and Tables

**Fig. 10:**
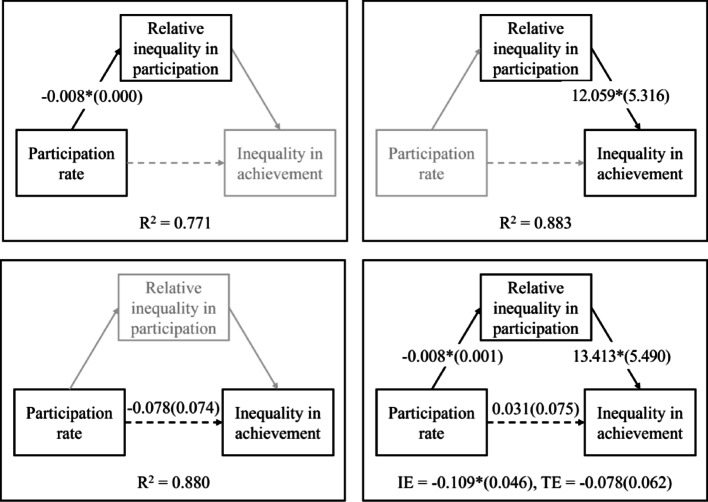
Results of the single paths and full mediation analyses in further analyses on relative inequality in ECE participation. *Note* Displayed are unstandardized coefficients from three separate country-fixed effects regression analyses and a country-fixed effects mediation model. Variables and paths that are printed grey were not included in the models. IE = indirect effect, TE = total effect. *p < .05

## Data Availability

The datasets analyzed during the current study are available in the PIRLS repository, https://www.iea.nl/data-tools/repository/pirls.

## Appendix 1: Item overview

See Tables 1 and 2.

**Table 1:** Overview of item wordings of the ECE participation variables across PIRLS cycles

Cycle	Item label	Item wording	Response categories
2001	ASBH0ATT	“Did your child attend <ISCED Level 0>?”	1 = *yes,* 2 = *no*
	ASBH0TIM	“If yes… How long was he/she in <ISCED Level 0>?”	1 = *more than 2 years,* 2 = *2 years,* 3 = *between 1 and 2 years,* 4 = *1 year,* 5 = *less than 1 year*
2006	ASBH0ATT	“Did your child attend <ISCED Level 0>?”	1 = *yes,* 2 = *no*
	ASBH0HL0	“If yes… How long was he/she in <ISCED Level 0>?”	1 = *3 years or more,* 2 = *between 2 and 3 years,* 3 = *2 years,* 4 = *between 1 and 2 years,* 5 = *1 year or less*
2011	ASBH04A	“Did your child attend <ISCED Level 0>?”	1 = *yes,* 2 = *no*
	ASBH04B	“If yes… How long was he/she in <ISCED Level 0>?”	1 = *3 years or more,* 2 = *between 2 and 3 years,* 3 = *2 years,* 4 = *between 1 and 2 years,* 5 = *1 year or less*
2016	ASBH05AA	“Did your child attend the following before <first grade>? Early childhood educational program or center for children under age 3”	1 = *yes,* 2 = *no*
	ASBH05AB	“Did your child attend the following before <first grade>? Pre-primary educational program for children age 3 or older, including <Kindergarten>”	1 = *yes,* 2 = *no*
	ASBH05B	“Approximately, how long was your child in these programs altogether?”	1 = *did not attend,* 2 = *less than 1 year,* 3 = *1 year,* 4 = *2 years,* 5 = *3 years,* 6 = *4 years or more*
2021	ASBH05AA	“Did your child attend the following before <first grade > ? Early childhood educational program or center for children under age 3”	1 = *yes,* 2 = *no*
	ASBH05AB	“Did your child attend the following before <first grade > ? Pre-primary educational program for children age 3 or older, including <Kindergarten > ”	1 = *yes,* 2 = *no*
	ASBH05B	“Approximately, how long was your child in these programs altogether?”	1 = *less than 1 year,* 2 = *1 year,* 3 = *2 years,* 4 = *3 years,* 5 = *4 years or more*

**Table 2:** Overview of item wordings of the parental education variables across PIRLS cycles

Cycle	Item label	Item wording	Response categories
2001	ASBHEDUF	“What is the highest level of education completed by the child’s father (or stepfather or male guardian) and mother (or stepmother or female guardian)? Child’s father”	1 = *some* <*ISCED Level 1 or 2*>*or did not go to school*, 2 = <*ISCED Level 2*>, 3 = <*ISCED Level 3A or 3B*>, 4 = <*ISCED Level 3C* >, 5 = <*ISCED Level 4A*>, 6 = <*ISCED Level 4B*>, 7 = <*ISCED Level 5A* >*or higher*, 8 = <*ISCED Level 5B* >*or higher*, 9 = *not applicable*
	ASBHEDUM	“What is the highest level of education completed by the child’s father (or stepfather or male guardian) and mother (or stepmother or female guardian)? Child’s mother”	1 = *some* <*ISCED Level 1 or 2*>*or did not go to school*, 2 = <*ISCED Level 2*>, 3 = <*ISCED Level 3A or 3B*>, 4 = <*ISCED Level 3C*>, 5 = <*ISCED Level 4A*>, 6 = <*ISCED Level 4B*>, 7 = <*ISCED Level 5A* >*or higher*, 8 = <*ISCED Level 5B* >*or higher*, 9 = *not applicable*
2006	ASBHLEDF	“What is the highest level of education completed by the child’s father (or stepfather or male guardian) and mother (or stepmother or female guardian)? Child’s father”	1 = *some* <*ISCED Level 1 or 2* > *or did not go to school*, 2 = <*ISCED Level 2*>, 3 = <*ISCED Level 3*>, 4 = <*ISCED Level 4*>, 5 = <*ISCED Level 5B*>, 6 = <*ISCED Level 5A, first degree*>, 7 = *beyond* <*ISCED Level 5A, first degree*>, 8 = *not applicable*
	ASBHLEDM	“What is the highest level of education completed by the child’s father (or stepfather or male guardian) and mother (or stepmother or female guardian)? Child’s mother”	1 = *some* <*ISCED Level 1 or 2* > *or did not go to school*, 2 = <*ISCED Level 2*>, 3 = <*ISCED Level 3*>, 4 = <*ISCED Level 4*>, 5 = <*ISCED Level 5B*>, 6 = <*ISCED Level 5A, first degree*>, 7 = *beyond* <*ISCED Level 5A, first degree*>, 8 = *not applicable*
2011	ASBH17A	“What is the highest level of education completed by the child’s father (or stepfather or male guardian) and mother (or stepmother or female guardian)? Child’s father”	1 = *did not go to school*, 2 = *some* <*ISCED Level 1 or 2*>, 3 = <*ISCED Level 2*>, 4 = <*ISCED Level 3*>, 5 = <*ISCED Level 4*>, 6 = <*ISCED Level 5B*>, 7 = <*ISCED Level 5A, first degree*>, 8 = *beyond* <*ISCED Level 5A, first degree*>, 9 = *not applicable*
	ASBH17B	“What is the highest level of education completed by the child’s father (or stepfather or male guardian) and mother (or stepmother or female guardian)? Child’s mother”	1 = *Did** not go to school*, 2 = *some* <*ISCED Level 1 or 2*>, 3 = <*ISCED Level 2*>, 4 = <*ISCED Level 3*>, 5 = <*ISCED Level 4*>, 6 = <*ISCED Level 5B*>, 7 = <*ISCED Level 5A, first degree*>, 8 = *beyond* <*ISCED Level 5A, first degree*>, 9 = *not applicable*
2016	ASBH18A	“What is the highest level of education completed by the child’s father (or stepfather or male guardian) and mother (or stepmother or female guardian)? Child’s father”	1 = *did not go to school*, 2 = *some* <*ISCED Level 1 or 2*>, 3 = <*ISCED Level 2*>, 4 = <*ISCED Level 3*>, 5 = <*ISCED Level 4*>, 6 = <*ISCED Level 5*>, 7 = <*ISCED Level 6*>, 8 = <*ISCED Level 7*>, 9 = <*ISCED Level 8*>, 10 = *not applicable*
	ASBH18B	“What is the highest level of education completed by the child’s father (or stepfather or male guardian) and mother (or stepmother or female guardian)? Child’s mother”	1 = *did not go to school*, 2 = *some* <*ISCED Level 1 or 2*>, 3 = <*ISCED Level 2*>, 4 = <*ISCED Level 3*>, 5 = <*ISCED Level 4*>, 6 = <*ISCED Level 5*>, 7 = <*ISCED Level 6*>, 8 = <*ISCED Level 7*>, 9 = <*ISCED Level 8*>, 10 = *not applicable*
2021	ASBH15A	“What it the highest level of education completed by the child’s <parents/guardians > ? <If the child has only one parent/guardian, answer for Parent/Guardian A. If there are two parents/guardians, choose one for Parent/Guardian A and the other for Parent/Guardian B. > <Parent/Guardian A > ”	1 = *did not go to school*, 2 = *some* <*ISCED Level 1 or 2*>, 3 = <*ISCED Level 2*>, 4 = <*ISCED Level 3*>, 5 = <*ISCED Level 4*>, 6 = <*ISCED Level 5*>, 7 = <*ISCED Level 6*>, 8 = <*ISCED Level 7*>, 9 = <*ISCED Level 8*>, 10 = *not applicable*
	ASBH15B	“What it the highest level of education completed by the child’s <parents/guardians > ? <If the child has only one parent/guardian, answer for Parent/Guardian A. If there are two parents/guardians, choose one for Parent/Guardian A and the other for Parent/Guardian B.> <Parent/Guardian B> ”	1 = *did not go to school*, 2 = *some* <*ISCED Level 1 or 2*>, 3 = <*ISCED Level 2*>, 4 = <*ISCED Level 3*>, 5 = <*ISCED Level 4*>, 6 = <*ISCED Level 5*>, 7 = <*ISCED Level 6*>, 8 = <*ISCED Level 7*>, 9 = <*ISCED Level 8*> , 10 = *not applicable*

## Appendix 2: Numerical results of trend analyses

See Table 3.

**Table 3:** Numerical results of trend analyses

	Trend in ECE participation rate in percent b(SE)	Trend in SES inequality in ECE participation b(SE)	Trend in SES inequality in reading achievement b(SE)
Intercept	85.679*(4.560)	5.093*(2.415)	63.564*(4.227)
2006	2.812(2.411)	2.608*(1.277)	2.222(2.235)
2011	8.003*(2.389)	− 0.215(1.265)	0.001(2.214)
2016	8.490*(2.400)	− 0.132(1.271)	1.701(2.225)
2021	9.888*(2.379)	− 0.157(1.260)	5.100*(2.205)
Country ID	included	included	included

Displayed are the results of three separate country-fixed effects trend analyses, regressing dependent variables on the PIRLS cycles and country IDs. The intercept reflects the estimate for Bulgaria in 2001. The country estimates are not displayed separately. **p* < .05

## Appendix 3: Results of mediation path models including country-fixed effects and control variables

See Table 4.

**Table 4:** Results of separate mediation path models including country-fixed effects and control variables

	Path A b(SE)	Path B b(SE)	Path C b(SE)
Path coefficient	− 0.312*(0.034)	0.268*(0.133)	− 0.059(0.070)
2006	4.455*(1.108)	2.044(2.289)	3.159(2.277)
2011	3.369*(1.138)	0.159(2.264)	0.880(2.340)
2016	3.866*(1.201)	2.903(2.408)	3.768(2.469)
2021	4.163*(1.275)	5.880*(2.549)	6.802*(2.622)
GDP per capita (z-standardized)	− 1.457(1.564)	− 2.912(3.189)	− 3.370(3.216)
Number of children (z-standardized)	6.439(5.525)	− 21.787(11.246)	− 20.197(11.359)
Net migration (z-standardized)	0.416(0.387)	2.737*(0.788)	2.883*(0.795)
Between-school segregation of high- and low-SES students (z-standardized)	1.217(0.693)	4.885*(1.406)	5.168*(1.424)
Mean age difference between high- and low-SES students (z-standardized)	− 1.325*(0.652)	0.525(1.343)	0.090(1.340)
Country ID	included	included	included
R^2^	0.863	0.905	0.903

Displayed are regression coefficients from three separate models. Path A = SES inequality in ECE participation regressed on ECE participation rate, PIRLS cycles, control variables, and country IDs. Path B = SES inequality in reading achievement regressed on SES inequality in ECE participation, PIRLS cycles, control variables, and country IDs. Path C = SES inequality in reading achievement regressed on ECE participation rate, PIRLS cycles, control variables, and country IDs. **p* < .05

## Appendix 4: Results of further analyses on longer ECE participation

See Figs. 7, 8 and 9.

## Appendix 5: Results of further analyses on relative inequality in ECE participation

See Fig. 10.

## Footnotes

1 Note that in this study and literature review, we focus on universal ECEC, not experimental programs such as the Abecedarian Program (Campbell & Ramey, 1994) that target at-risk children.

2 Note that due to the Covid-19 pandemic, the data collection was partly delayed in the 2021 cycle.

3 Note that some countries participate separately with different strata (e.g., Belgium (Flemish) and Belgium (French)). For the sake of simplicity, we refer to these as countries in the text.

4 Among the other international large-scale assessments that contain student achievement tests, some have only three measurement cycles so far (e.g., International Computer and Information Literacy Study (ICILS), International Civic and Citizenship Education Study (ICCS)). In TIMSS, the population B assessment does not contain information on prior ECE participation in the available cycles, and in the population A assessment only since 2011. PISA contains student-reported information on ECE participation since 2009, but this item was changed considerably in 2018.

5 Note that countries can deviate from this international target grade.
